# Comprehensive discovery and characterization of small RNAs in *Corynebacterium glutamicum* ATCC 13032

**DOI:** 10.1186/1471-2164-14-714

**Published:** 2013-10-19

**Authors:** Almut Mentz, Armin Neshat, Katharina Pfeifer-Sancar, Alfred Pühler, Christian Rückert, Jörn Kalinowski

**Affiliations:** Microbial Genomics and Biotechnology, Center for Biotechnology, Bielefeld University, Universitätsstraße 27, 33615 Bielefeld, Germany; Senior Research Group Genome Research of Industrial Microorganisms, Center for Biotechnology, Bielefeld University, Universitätsstraße 27, 33615 Bielefeld, Germany; Technology Platform Genomics, Center for Biotechnology, Bielefeld University, Universitätsstraße 27, 33615 Bielefeld, Germany

**Keywords:** Bacteria, Non-coding RNA, High-throughput sequencing, RNA regulation

## Abstract

**Background:**

Recent discoveries on bacterial transcriptomes gave evidence that small RNAs (sRNAs) have important regulatory roles in prokaryotic cells. Modern high-throughput sequencing approaches (RNA-Seq) enable the most detailed view on transcriptomes offering an unmatched comprehensiveness and single-base resolution. Whole transcriptome data obtained by RNA-Seq can be used to detect and characterize all transcript species, including small RNAs. Here, we describe an RNA-Seq approach for comprehensive detection and characterization of small RNAs from *Corynebacterium glutamicum*, an actinobacterium of high industrial relevance and model organism for medically important *Corynebacterianeae*, such as *C. diphtheriae and Mycobacterium tuberculosis*.

**Results:**

In our RNA-Seq approach, total RNA from *C. glutamicum* ATCC 13032 was prepared from cultures grown in minimal medium at exponential growth or challenged by physical (heat shock, cold shock) or by chemical stresses (diamide, H_2_O_2_, NaCl) at this time point. Total RNA samples were pooled and sequencing libraries were prepared from the isolated small RNA fraction. High throughput short read sequencing and mapping yielded over 800 sRNA genes. By determining their 5′- and 3′-ends and inspection of their locations, these potential sRNA genes were classified into UTRs of mRNAs (316), *cis*-antisense sRNAs (543), and *trans-*encoded sRNAs (262). For 77 of *trans*-encoded sRNAs significant sequence and secondary structure conservation was found by a computational approach using a whole genome alignment with the closely related species *C. efficiens* YS-314 and *C. diphtheriae* NCTC 13129. Three selected *trans-*encoded sRNAs were characterized by Northern blot analysis and stress-specific transcript patterns were found.

**Conclusions:**

The study showed comparable numbers of sRNAs known from genome-wide surveys in other bacteria. In detail, our results give deep insight into the comprehensive equipment of sRNAs in *C. glutamicum* and provide a sound basis for further studies concerning the functions of these sRNAs.

**Electronic supplementary material:**

The online version of this article (doi:10.1186/1471-2164-14-714) contains supplementary material, which is available to authorized users.

## Background

*Corynebacterium glutamicum* is a non-pathogenic and non-sporulating gram-positive soil bacterium which belongs to the order Actinomycetales. This microorganism has a long history of applications in the production of various amino acids and other industrially relevant compounds [1, 2]. Furthermore, it serves as a model organism for close relatives with medical significance such as *C. diphtheriae or Mycobacterium tuberculosis*. The genome sequence established a decade ago [3, 4] comprises a circular chromosome with a length of almost 3.3 Mb and harbors more than 3000 annotated protein-coding sequences (CDS). Based on the complete genome sequence, transcriptional regulation in *C. glutamicum* has been studied extensively [5] and revealed a complex regulatory network including 97 transcriptional regulator proteins with so far 1443 regulatory interactions [6]. However, only very little is known about small RNAs (sRNA) and their potential regulatory actions in this organism. Information on RNA species beside ribosomal RNA (rRNA) or transfer RNA (tRNA) is absent from the current genome annotation. It can only be deduced from the genome sequence that *C. glutamicum* lacks a sequence homologue of the RNA chaperone Hfq, similar to other *Actinomycetales*[7]. So far, the only experimentally defined sRNA in *C. glutamicum* (ArnA) was detected upstream of the GntR-Regulator *cg1935* and is located in antisense direction [8].

Recently, regulatory RNAs have been detected in all three domains of life with unexpectedly large numbers, in the range of hundreds per bacterial and thousands per eukaryotic genome. In most cases, these transcripts do not encode proteins and so the term non-coding RNA (ncRNA) is often applied synonymously. All hitherto identified RNA families are collected in the RNA-families (Rfam) online database [9], fRNAdb [10], and sRNAdb [11]. Beyond *trans*-encoded sRNA genes, these databases also include RNA motifs from mRNA leader transcripts of protein-coding genes, some of which regulate translation initiation or cause transcriptional attenuation. Elements such as RNA thermometers are structures sensitive to temperature shifts and control the accessibility of the Shine-Dalgarno sequence of the mRNA leader (reviewed in [12]). The classes of attenuation mechanisms are diverse and include amongst others small molecule-mediated riboswitches (reviewed in [13]) as well as classical attenuators regulated by translation of a small leader peptide.

The length of bacterial sRNAs is generally between 50 and 300 nt [14] and can be up to 500 nt [15]. In addition to RNAs with housekeeping function, in-depth analyses of several sRNAs led to the discovery of various novel regulatory functions. These functions modulate a wide range of responses to stresses and other environmental stimuli (reviewed in [16]) including RNA processing and RNA degradation as well as translation control. Different mechanisms of action have been described, the majority representing interactions through basepairing between sense RNA and regulating antisense RNA. Direct transcriptional regulation through sRNAs seems to occur rarely, and was first discovered for 6S RNA of *E. coli*[17]. The much more frequent post-transcriptional regulation by *trans*-encoded sRNAs works through imperfect basepairing with target mRNAs (reviewed in [16]). These sRNAs show stable secondary structures and their genes are generally located in the “intergenic regions” between protein-coding sequences. In contrast, *cis*-antisense sRNAs (asRNA) genes are located directly in the antisense direction with respect to their target genes and thus show full complementarity (reviewed in [18]).

To date, different strategies have been applied for the systematic genome-wide search for sRNAs. In the enterobacterium *E. coli*, a number of sRNAs have been predicted by computational methods (reviewed in [19]). Such *in silico* analyses are usually based on common features of sRNAs such as thermodynamic stability, structure conservation, or sequence similarity between species [20], as well as the existence of Rho-independent terminators at their 3′-ends [21]. The Rfam database provides sRNA predictions for organisms with known genome sequences calculated from sequence covariance models. In *C. glutamicum*, four sRNAs are predicted by Rfam, including 6C RNA and the housekeeping RNAs tmRNA, RNAse P, and SRP/4.5S RNA*.*

Experimental strategies for the discovery of sRNAs in bacteria started with systematic genome-wide screens by shotgun cloning and sequencing of cDNA [22] or by using tiling microarrays (reviewed in [23]) and detected large numbers of sRNAs in all tested organisms. Undoubtedly, new high-throughput sequencing techniques enable the most detailed view on a cellular transcriptome. Thus, RNA-sequencing has emerged as a powerful tool for the detection of bacterial sRNAs [24–26]. The creation of RNA-sequencing libraries can vary between different platforms in high throughput sequencing [27] but there are similarities between the procedures. An important step to increase the coverage of mRNA or sRNA in transcriptome sequencing data is the depletion of highly abundant ribosomal RNAs. Another is to ensure that the strand-information of the RNA is kept in the cDNA sequence. This can be done by using adapters of known sequence to be ligated to the RNA before cDNA synthesis. In addition, various specific enzymatic treatments of the RNA samples can be used for mapping of transcriptional starts [26] or for detection of processing sites [28].

Here, we present the first deep sequencing study of sRNAs in *C. glutamicum*. Sequencing libraries were created by the “differential” RNA-sequencing (dRNA-Seq) approach [26] with RNA samples from exponential growth phase and stress conditions such as heat and cold shock, salt stress, H_2_O_2_ and diamide stress to gain a broad spectrum of transcription of potential sRNA genes in response to these conditions. Supported by promoter searches, RNA-Seq data were analyzed and led to the detection of novel sRNA genes in *C. glutamicum* ATCC 13032. In addition, sRNA genes were classified and compared with bioinformatic sRNA predictions based on secondary structure stability and sequence conservation.

## Results

### Detection of potential sRNA genes in *C. glutamicum* ATCC 13032 by transcriptome sequencing and read-mapping

**Figure 1:**
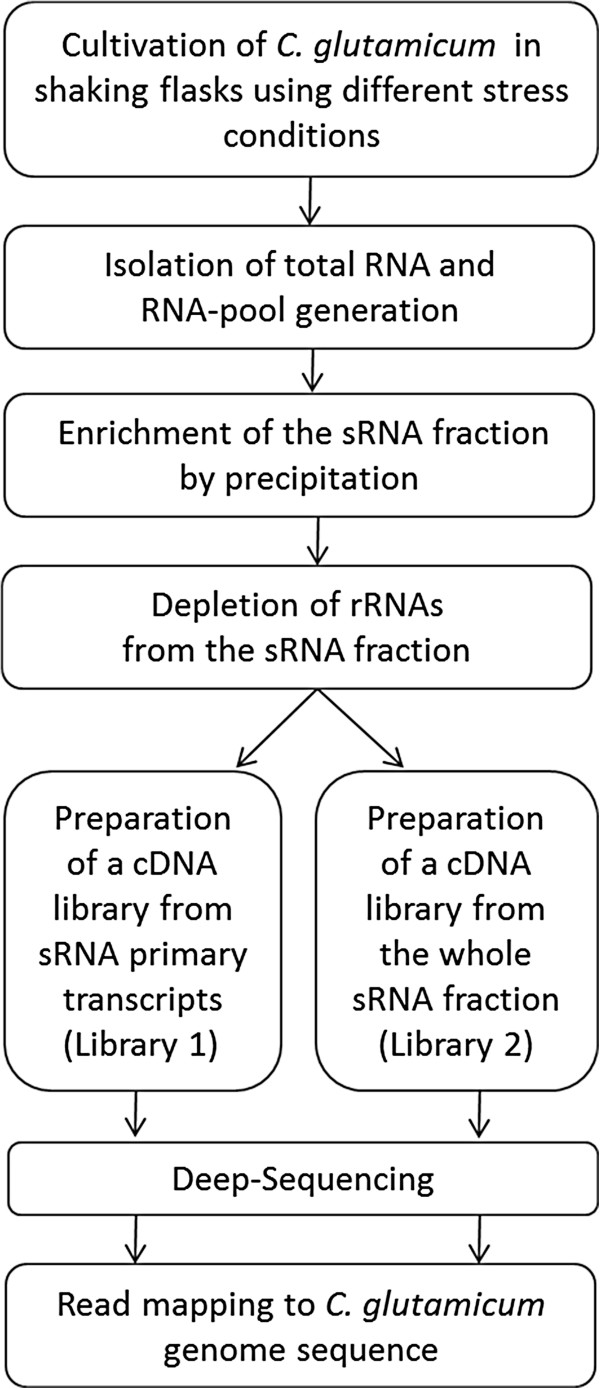
**Workflow for a small RNA-Seq approach in** ***C. glutamicum*** **ATCC 13032.** Before cDNA library preparation, the small RNA fraction was split into two samples for creation of two different sequencing libraries. The first sample was treated with a 5′-monophosphate-specific exonuclease to degrade transcripts that are processed or undergoing degradation. The second sample was left untreated and represents the whole of sRNA transcripts within the cell. After cDNA library preparation, both samples were then separately committed to strand-specific high-throughput sequencing.

Transcription of sRNAs in bacteria is highly variable under different environmental conditions [16, 29]. Hence, for a comprehensive survey of sRNAs in *C. glutamicum*, we isolated total RNA from *C. glutamicum* cells grown to exponential phase and from cells after a variety of stress treatments and pooled the total RNA samples (Figure 1). The stress treatments were heat shock (50°C), cold shock (10°C), oxidative stress (1% H_2_O_2_), diamide stress (2 mM), and salt stress (1.5 M NaCl). To enrich small RNA for a transcriptome sequencing (RNA-Seq), the pool of total RNAs was size-selected for transcripts smaller than 250 nucleotides (nt) by precipitation and further depleted of ribosomal RNAs (rRNA) using a hybridization procedure that selectively binds rRNA species with biotinylated probes (Figure 1). The probe:rRNA hybrids were then captured by magnetic beads and removed using a magnet. After this step, the sRNA fraction was split into two samples as in the differential RNA sequencing (dRNA-Seq) approach [26]. Hereby, one sample was treated with a 5′-monophosphate-specific exonuclease which degrades specifically transcripts that are processed or undergoing degradation, thus leaving primary transcripts with native 5′-triphosphate ends. The second sample was left untreated as a representation of the whole small transcriptome of the cell. The small RNA samples were then separately committed to strand-specific sequencing library preparation using the standard Illumina TruSeq Small RNA kit. Both cDNA libraries were sequenced on an Illumina GA *IIx* sequencer, obtaining 35 bases long single reads from their 5′-ends. Reads were mapped to the chromosome sequence of the *C. glutamicum* ATCC 13032 wild-type strain [3] using the SARUMAN algorithm implemented in CUDA programming language and run on computer graphics cards [30] allowing for up two mismatches per read. In total, 7,869,859 reads were uniquely mapped for the primary transcripts sample (library 1; Table 1) and 22,752,379 reads were uniquely mapped in case of the total small RNA sample (library 2; Table 1). The rRNA was found to be more efficiently depleted in the enzyme-treated library 1, yielding a proportion of only 3% of total reads mapping to ribosomal RNA genes. It also became apparent that the enzyme treatment had depleted residual mRNA as seen by the lower fraction of reads attributable to the sense direction of CDS. The remaining set of reads were mapped either *cis*-antisense to CDS or to regions with no annotated genome features (potential sRNA fraction). The *cis*-antisense reads made up 3% and less in both libraries. Interestingly, the majority of read mappings belong to regions with no annotated features, particularly in the library enriched for primary transcripts (59%), indicating a high number of potential sRNAs in *C. glutamicum*.

**Table 1:** **Distribution of mapped reads to annotated features in the** ***C. glutamicum*** **13032 genome in two different sequencing libraries**

Annotated feature category	sRNA primary transcripts^a^(library 1)		Whole sRNA transcripts (library 2)	
	Reads	[%]	Reads	[%]
rRNA genes	255,591	3.2	5,230,021	23.0
tRNA genes	1,050,962	13.4	2,130,360	9.3
mRNA genes (CDS, sense)	1,686,575	21.4	7,134,344	31.4
**Potential sRNA fraction** (remaining set of mapped reads)				
*cis*-antisense (CDS, antisense)	242,429	3.1	450,589	2.0
Regions with no annotated features	4,634,302	58.9	7,807,065	34.3
Total	7,869,859		22,752,379	

^a^sRNA primary transcripts were obtained using a 5′-monophosphate-specific exonuclease which degrades specifically transcripts that are either processed or undergoing degradation.

### Characterization of potential sRNA genes with the help of bioinformatic promoter analysis

**Figure 2:**
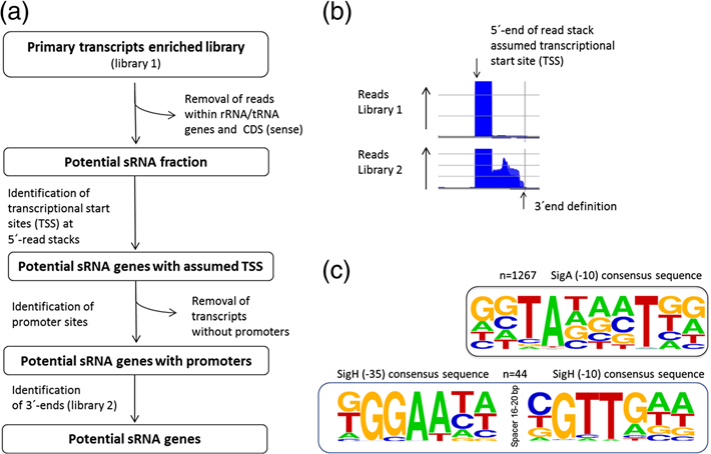
**Definition of start and stop positions of potential sRNA genes. (a)** Workflow for characterization of sRNA genes with the help of bioinformatic promoter analysis at transcriptional start sites (TSS) in library 1 (primary transcripts enriched). All TSS without promoters were removed and the 3′-ends of potential sRNA genes were determined with sequence data from library 2. **(b)** Library 1 (primary transcripts enriched) was used for definition of transcriptional start sites at 5′-ends of read stacks. The 5′-end of a read stack is defined as a number of read starts that exceed the number of read starts at the previous position by a factor of 20. **(c)** Weblogo [37] presentations of the consensus sequences of -35 and -10 core regions. In detail, 1267 SigA and 44 SigH promoter sites were detected by the Improbizer tool. The percentage of occurrence of a nucleotide at a particular position is represented by the size of the nucleotide symbol (A, C, G, T).

After filtering of mappings to tRNA and rRNA genes and to putative mRNAs (inside CDS, sense direction), the potential sRNA fraction from the library 1 (primary transcripts enriched) was utilized for the definition of sRNA transcript starts (Figure 2a). A number of reads that start at a distinct genomic position normalized to the previous position was defined as read stack and a transcriptional start site (TSS) was assumed at the 5′-position at each of these stacks (Figure 2b). For the experiment performed here, the number of read starts used in stringent filtering was determined to be 20. This analysis yielded a number of 2899 stacks (1304 stacks *cis*-antisense to CDS and 1595 stacks in regions lacking annotated features). As a further filtering step, the 5′-upstream sequences of the assumed TSS were analyzed for promoters. Using the tool Improbizer [31, 32], we searched for matches to the consensus promoter sequences recognized by the primary housekeeping sigma factor SigA [33] or the stress-related ECF-family sigma factor SigH [34], which is known to play a major role under oxidative stress [35] and heat stress conditions [36]. Thus, 1267 putative TSS were found to exhibit an upstream SigA-recognized promoter sequence (Figure 2c) (531 *cis*-antisense to CDS and 736 SigA-dependent promoters in regions lacking annotated features). The search for SigH promoter sequences was successful in case of 44 TSS (11 *cis*-antisense to CDS and 33 in regions lacking annotated features). For 14 transcripts, promoters of both types were detected, indicating sRNA variants with different 5′-ends.

To determine the 3′-ends of sRNAs, we mapped reads from the two libraries and followed each of the initial stacks up to a point where the number of read starts fell below the chosen cut-off of 10 reads and defined 3′-ends from these data (Figure 2c). In addition, we searched for Rho-independent transcription terminators since these have been reported for numerous of bacterial sRNAs [38]. This search was performed with the tool TransTermHP [39] targeting thymine-rich stretches of DNA following a hairpin loop within 60 nt around the assumed 3′ends. Thereby, Rho-independent terminators were found at 69 of these sRNAs (4 *cis*-antisense to CDS and 65 in regions lacking annotated features).

In 136 cases, the predicted TSS were within close distance to each other, indicating multiple promoters. Proposed multiple starts located within 100 bp at the 5′-end and with the same 3′-end were merged to a single region and annotated as such.

### Classification of potential sRNA genes by their positions relative to annotated protein-coding sequences

**Figure 3:**
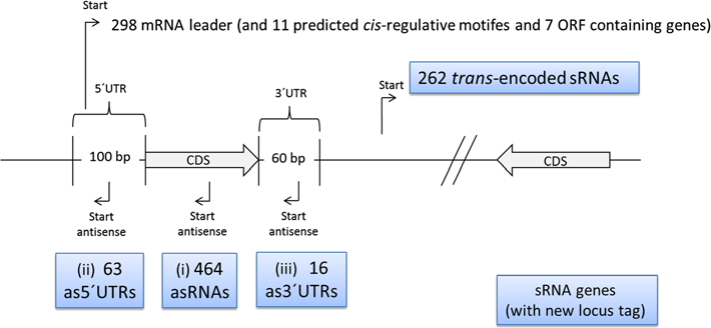
**Classification of potential sRNA genes by their positions relative to annotated protein-coding sequences.** Transcripts with a downstream CDS in less than 100 nt distance from their 3′-ends were designated as “mRNA leader”. The class also includes riboswitches and ORF containing transcripts already predicted and stored in the Rfam database [9]. The class “antisense transcripts” comprises three sub-types (i) *cis*-antisense RNAs (asRNA) that start within a CDS, (ii) transcripts antisense to a 5′-UTR, starting within 100 nt from the 5′-end of a CDS (as5′-UTR) and (iii) transcripts antisense to a 3′-UTR, starting within 60 nt from the 3′-end of a CDS (as3′-UTR). All remaining intergenic transcripts were categorized as *trans*-encoded sRNAs.

In order to identify putative untranslated regions (UTRs) of mRNAs that are included in the set of potential sRNA genes, these were then grouped according to their position and direction relative to an adjacent CDS (Figure 3). In total, 298 transcripts that had a downstream CDS in less than 100 nt distance from their 3′-ends were designated as “mRNA leader”, (Additional file 1). By comparison with the RNA-families database (Rfam [9]) we could additionally assign the well conserved *mraW* motif (at *cg2377*), the *cspA* motif (at *cg0215*), and nine predicted riboswitches which are part of 5′-UTRs longer than 100 nt (Additional file 2). This way we validated the Rfam-predicted thiamine pyrophosphate (TPP)-dependent riboswitches upstream of genes from the thiamine biosynthesis pathway, ThiC (*cg1476*), ThiM (*cg1655*), and ThiE (*cg2236*). Two TPP-riboswitches were found upstream of *cg0825* (putative beta-ketoacyl acyl carrier protein reductase) and *cg1227* (putative membrane protein). One flavin mononucleotide (FMN)-dependent riboswitch was detected at the putative nicotinamide mononucleotide uptake permease (*cg0083*) and one S-adenosyl methionine (SAM)-dependent type IV riboswitch upstream of *cg1478* (annotated as hypothetical protein). Together with the two riboswitch related RNA-motifs (both *yybP-ykoY*) [40] and seven additional transcripts putatively encoding small proteins, we ended up with 316 regions in the class “mRNA leader”.

The class “antisense transcripts” comprises 543 regions (Additional file 3) and includes three sub-types (i) *cis*-antisense RNAs (asRNA) that start in antisense orientation within an opposing CDS, (ii) transcripts antisense to a 5′-UTR, starting within 100 nt from the 5′-end of an opposing CDS (as5′-UTR) and (iii) transcripts antisense to a 3′-UTR, starting within 60 nt from the 3′-end of an opposing CDS (as3′-UTR). This analysis defined 464 sRNA regions as asRNAs, 63 as as5′-UTRs, and 16 as as3′-UTRs. Eight as5′-UTRs were also counted as3′-UTRs and 48 mRNA leader were also counted as5′-UTRs due to special arrangements of CDS. All remaining 262 regions were designated as *trans-*encoded sRNAs (Additional file 4). For preparation of an updated *C. glutamicum* genome annotation, only antisense transcripts and *trans*-encoded RNAs were assigned with locus-tags. In the new nomenclature the locus tag of each CDS will be extended by a trailing zero (e.g. old: *cg0001;* new: *cgb_00010*). The last digit of the number is used to number novel features in between of old features.

### Detection of small *C. glutamicum* genes encoding small proteins

Some of the sRNAs might actually represent mRNAs and encode small proteins. We applied the “ORFfinder” online tool to extract ORFs from sequences in multiple FASTA format and subsequently searched for ribosomal binding sites (RBSs) upstream of the extracted ORF sequences with RBSfinder [41] using a window size of 15 bp and the standard RBS settings. Only ORFs with a minimum length of 15 amino acid residues showing either a RBS (4) or leaderless transcripts (4) were taken into account, and in total eight small mRNAs were retrieved (Additional file 5). Next, we searched for conservation of these peptide sequences in other bacterial genomes using the TBlastX algorithm at the NCBI web portal (http://blast.ncbi.nlm.nih.gov/Blast.cgi) and found conservation in seven cases (Additional file 5). Similar small proteins with E-values less than 10^-4^ were found in other *Corynebacterium* species and also outside Corynebacteria. More widely conserved proteins comprise the well conserved peptide-tag encoded by the tmRNA that was identified with 12 amino acids length (AEKSQRDYALAA) in *C. glutamicum*. Beside the peptide encoded by tmRNA, only one other peptide, *cgb_08775* (*cg4014*), was detected to be conserved in species beyond *Corynebacterium.* The smallest of all conserved peptides with 15 amino acids length was found to be *cgb_14345* (*cg4016*). This peptide is already known as valine-containing leader peptide in front of the *ilvBNC* operon [42]. Further putative leader peptides of attenuator structures were detected at *cgb_33575* (*cg4012*) located upstream of *trpE* of the tryptophan operon, at *cgb_03035* (*cg4015*) in front of the *leuA* gene (*cg0303*), encoding isopropylmalate synthase, the first step in leucine biosynthesis, and at *aroF* (*cg1129*). Supporting their functional assignment is the occurrence of three consecutive tryptophan residues in the putative leader peptide upstream of the tryptophan operon, four consecutive leucine codons in the presumed *leuA* leader peptide and the amino acids phenylalanine-tyrosine-phenylalanine in the case of the *aroF* leader peptide.

### Analysis of *cis*-antisense RNA genes, located within protein-coding genes

**Figure 4:**
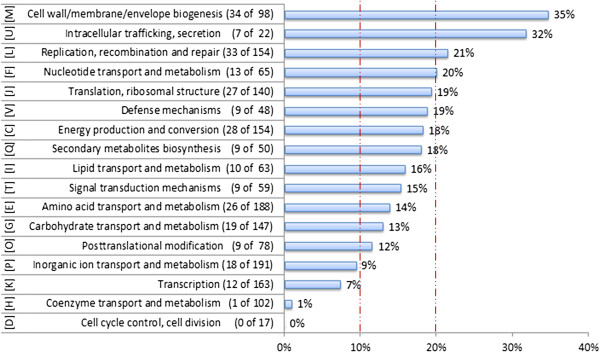
**Functional classification of** ***C. glutamicum*** **genes having** ***cis*** **-antisense RNAs (asRNAs) according to eggNOG.** Occurrences and frequencies of asRNAs at genes with assigned eggNOG [43] classes (264 of 464) are shown. Classes with *cis*-antisense RNA frequencies between 10% and 20% reflect a normal distribution. Red lines represent limits for classes that are underrepresented (<10%) or overrepresented (>20%).

In our study, more than half of the sRNA regions (543 of 807) fall into the class “antisense transcripts” (Figure 3, Additional file 3). For the sub-type of asRNAs (464), which are located directly opposite to a CDS, the mean length was calculated to be 55 nt. This very small size particularly for asRNAs is shown in a box plot diagram (Additional file 6). The asRNAs are distributed to 409 different CDSs with 44 CDS having more than one antisense transcript. It is noteworthy that antisense transcription is not only observed at the 5′-ends of the corresponding CDS, but often also at the 3′-ends or in the middle of a coding region. To correlate the functions of encoded proteins with observed asRNAs, the respective proteins were classified according to the eggNOG functional classification system [43]. Thereby, 264 of 464 asRNAs match to CDS with eggNOG classification (Figure 4). In case of the remaining 200 asRNA, either no category or categories with poor characterization were retrieved. A normal distribution of 464 asRNAs over all currently annotated CDS would result in about 15% of the genes of each eggNOG class to contain a asRNA. We considered classes containing a asRNA in more than 20% or less than 10% of the members of the class as over- or underrepresented. The lowest proportions of asRNAs were observed for genes within the classes’ cell cycle control/cell division (“D”, 0%), coenzyme transport and metabolism (“H”, 1%), transcription (“K”, 7%), and inorganic transport and metabolism (“P”, 9%). On the other hand, we observed a higher proportion of asRNAs within genes from the functional class cell envelope biogenesis (“M”, 35%). Moreover, a higher proportion of genes which are involved in secretion processes (“U”, 32%) seem to have transcription in antisense direction (Figure 4). Among the genes involved in protein secretion there were both protein secretion mechanisms represented, with *secY* (*cg0647*) encoding a preprotein translocase subunit of the Sec secretion system and *tatC* (*cg1684*) encoding a twin-arginine (Tat) secretion translocase subunit. The *tatC* antisense RNA (*cgb_16835*) is apparently transcribed from a SigA-dependent promoter, whereat the *secY* asRNA (*cgb_06475*) seems to be transcribed from a SigH-controlled promoter.

In this context, we tested also the proportions of asRNAs at genes encoding transmembrane helices or signal peptides for secretion. By bioinformatic search 165 of 464 asRNAs (~35%) were detected at the corresponding genes. Statistically, this proportion is not significantly different from a normal distribution since 974 genes (~ 32% of all annotated *C. glutamicum* genes) encode a signal peptide for secretion or at least one transmembrane helix.

The occurrence of asRNAs includes also two of the 13 two-component regulatory systems in *C. glutamicum,* namely the sensory histidine kinase genes *cgtS4* (*cg0483*) and *cgtS6* (*cg3060*) that sense a specific environmental stimulus at the membrane and the corresponding response regulators genes *cgtR4* (*cg2888*) and *cgtR6* (*cg3061*) that mediate the transcriptional regulation by binding to operators [44]. The *cgtSR4* genes are involved in phosphate starvation [45] and *cgtR4* seems to be essential [46]. Further asRNAs are located opposite to transcriptional regulator genes (within eggNOG class K) such as *cysR* (*cg0156*) and *sufR* (*cg1756)* which are involved in assimilatory sulphate reduction [47]and thiol-oxidative stress defense [48], respectively. Beside this, we detected asRNAs at the *acnR* gene (*cg1738*) [49] and other members of the TetR family (*cg2686; cg1308*). The following transcriptional regulators were also identified to have antisense transcription, SugR (*cg2115*) as regulator of the PEP:sugar phosphotransferase system genes [50, 51], NdrR (*cg2112*) the regulator of deoxyribonucleotide reduction [52], PcaO (*cg2627)* the transcriptional activator of the ketoadipate metabolism genes [53], FarR (*cg3202*) a transcriptional regulator involved in nitrogen metabolism [54], and two members of the HTH_3-family (*cg1392; cg2040*).

### Bioinformatic analysis of sequence and structural conservation of *trans*-encoded sRNAs in *C. glutamicum* ATCC 13032

In bacteria sRNAs often have characteristic structures that are conserved stronger in evolution than their primary sequences. Hence, structure conservation analysis is integrated in a number of sRNA prediction tools. In our approach, we used the RNAz tool [20] to detect secondary structure conservation in a multiple genome alignment between the closely related species of *C. glutamicum* ATCC 13032, *C. efficiens* YS-314 and *C. diphtheriae* NCTC 13129. RNAz predictions made under stringent conditions (p ≥ 0.9) overlap with 45 *trans*-encoded sRNA genes detected with RNA-Seq (Table 2). Moreover, the sequencing results were compared with a less stringent set of RNAz predictions (p ≥ 0.5) which resulted in 77 of 262 *trans-*encoded sRNAs matching to loci predicted by RNAz (~ 30%) (marked in Additional file 4). Hereby, all three housekeeping RNAs, tmRNA *(cgb_09183*), M1 RNA (*cgb_24535*), and 4.5S RNA (*cgb_02933*) were predicted at positions very similar to those of the Rfam database entries that were calculated by covariance models [9]. This was the case also for 6C RNA (*cgb_03605*), which is known to be present in many *Actinomycetales* genera [55]. The 6C RNA was named from its two stem-loops, each typically containing six cytosine (C) residues. Interestingly, in *C. glutamicum* the 6C RNA has two stretches of eight cytosines. However, the function of these cytosine homopolymers is not known and therefore the relevance of this difference is unclear.

**Table 2:** ***Trans*** **-encoded sRNA genes with overlapping RNAz- prediction (p ≥ 0.9) and their prediction details**

New locus tag	Strand	Sequencing start	Sequencing stop	Adjacent genes	RNAz Prediction start	RNAz Prediction end	RNAz max. p-score
*cgb_00105*	-	10053	9921	*cg0010(-)/cg0012(-)*	10073	9921	0.95
*cgb_00925*	+	74286	74320	*cg0092(+)/cg0095(+)*	74297	74476	0.99
*cgb_03505*	-	307582	307548	*cg0350(-)/cg0352(-)*	307558	307474	0.95
^*a*^ *cgb_03605*	+	314679	314787	*cg0360(-)/cg0362(+)*	314611	314792	0.99
*cgb_03995*	-	346945	346882	*cg0399(-)/cg0400(-)*	346922	346590	0.96
*cgb_05085*	+	452359	452408	*cg0508(-)/cg0510(+)*	452321	452622	0.99
*cgb_05716*	+	509744	509990	*cg0571(+)/cg0572(+)*	509724	509981	0.99
*cgb_05756*	+	512702	512814	*cg0575(+)/cg0576(+)*	512744	512906	0.99
*cgb_08496*	+	782757	782836	*cg0849(+)/cg0850(+)*	782647	782889	0.99
*cgb_08785*	-	807467	807331	*cg0878(-)/cg0879(+)*	807563	807292	0.92
*cgb_09095*	-	842812	842715	*cg0909(-)/cg0910(-)*	842945	842791	0.97
*cgb_09097*	-	842983	842911	*cg0909(-)/cg0910(-)*	842945	842791	0.97
^*b*^ *cgb_09185*	+	848500	848922	*cg0918(+)/cg0919(+)*	848444	848993	0.90
*cgb_09483*	+	878863	878996	*cg0948(-)/cg0949(+)*	878852	879125	0.99
*cgb_13305*	-	1237440	1237333	*cg1330(+)/cg1332(-)*	1237507	1237208	0.90
*cgb_14495*	+	1351975	1352041	*cg1449(+)/cg1451(+)*	1351833	1352246	0.99
*cgb_17355*	-	1626662	1626596	*cg1735(-)/cg1736(-)*	1626885	1626583	0.95
*cgb_17735*	+	1665705	1665791	*cg1773(-)/cg1774(+)*	1665657	1665835	0.99
*cgb_17805*	-	1672717	1672673	*cg1780(+)/cg1781(-)*	1672744	1672565	0.92
*cgb_18405*	-	1734383	1734304	*cg1840(-)/cg1841(-)*	1734440	1734264	0.99
*cgb_18415*	-	1736390	1736347	*cg1841(-)/cg1842(+)*	1736478	1736333	0.96
*cgb_21516*	-	2039580	2039466	*cg2151(-)/cg2152(-)*	2039656	2039493	0.98
*cgb_21673*	-	2055867	2055764	*cg2167(-)/cg2168(-)*	2055929	2055750	0.99
*cgb_22185*	-	2108839	2108800	*cg2218(-)/cg2221(-)*	2109027	2108748	0.98
*cgb_22215*	-	2110108	2109924	*cg2221(-)/cg2222(-)*	2110102	2109913	0.99
*cgb_22285*	-	2116294	2116236	*cg2228(-)/cg2229(-)*	2116297	2115998	0.96
*cgb_22405*	-	2124418	2124384	*cg2240(+)/cg2241(-)*	2124504	2124285	0.92
*cgb_23783*	-	2267593	2267546	*cg2378(-)/cg2380(-)*	2267720	2267551	0.93
*cgb_24455*	-	2331195	2331116	*cg2445(-)/cg2446(-)*	2331257	2331109	0.99
^*c*^ *cgb_24535*	-	2343003	2342592	*cg2453(-)/cg2455(-)*	2343050	2342650	0.95
*cgb_24775*	-	2362678	2362640	*cg2477(-)/cg2478(-)*	2362704	2362548	0.94
*cgb_25636*	+	2447380	2447441	*cg2563(+)/cg2564(-)*	2447231	2447490	0.94
*cgb_25955*	-	2476453	2476419	*cg2595(-)/cg2597(-)*	2476484	2476295	0.92
*cgb_26475*	-	2530082	2530004	*cg2647(-)/cg2648(+)*	2530150	2529972	0.91
*cgb_28315*	+	2693243	2693292	*cg2831(-)/cg2833(+)*	2692958	2693349	0.99
*cgb_28685*	-	2730160	2730126	*cg2868(+)/cg2869(-)*	2730432	2730065	0.97
*cgb_29606*	-	2816731	2816535	*cg2960(+)/cg2962(-)*	2816711	2816532	0.99
*cgb_30116*	-	2863994	2863960	*cg3011(-)/cg3012(-)*	2864045	2863846	0.95
*cgb_30685*	-	2928726	2928634	*cg3068(-)/cg3069(-)*	2928804	2928585	0.99
*cgb_31375*	-	2997519	2997485	*cg3137(-)/cg3138(+)*	2997679	2997500	0.94
*cgb_31785*	-	3041252	3041154	*cg3178(-)/cg3179(-)*	3041427	3041124	0.97
*cgb_33045*	-	3156331	3156287	*cg3304(-)/cg3306(-)*	3156621	3156254	0.91
*cgb_33325*	+	3179928	3180066	*cg3332(-)/cg3334(+)*	3179728	3180003	0.99
*cgb_34325*	-	3282122	3282086	*cg3432(-)/cg3434(+)*	3282124	3281877	0.95

^a^RF01066; 6C.

^b^RF00023; tmRNA.

^c^RF00010; RNaseP.

### Experimental validation of three *trans-*encoded sRNAs by Northern blotting of stress-specific RNA samples and detailed structure analysis

**Figure 5:**
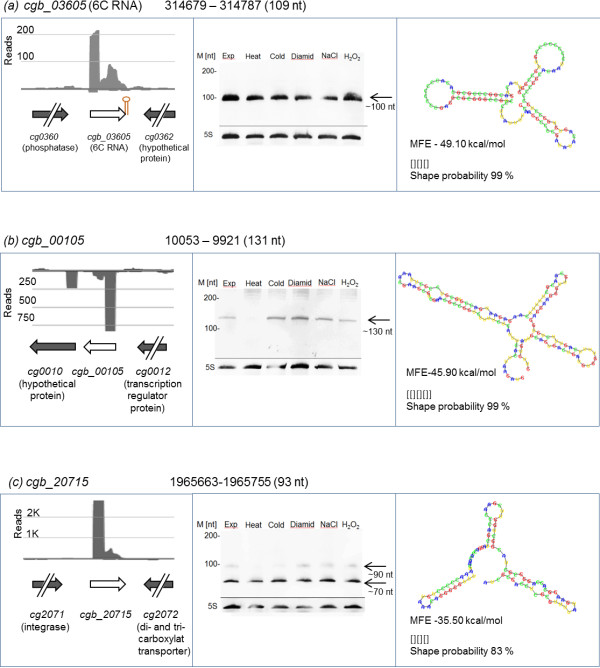
**Secondary structure prediction and experimental validation by Northern hybridizations of three selected sRNAs.** For each of the three sRNA genes, the left column shows the sequence coverage profile derived from library 1 (grey color). The y- and x-axis represent coverage and sequence localization. Grey arrows represent flanking genes, white arrows represent the sRNAs and brown stem-loop structures represent Rho-independent terminators. The middle column displays Northern blot results of all tested conditions at time point of harvesting of *C. glutamicum* cells, respectively. Exp = Exponential phase (OD_600_ 10), unstressed. Further conditions were heat, cold, diamide, NaCl, and H_2_O_2_ stresses, all applied for 15 minutes at an OD_600_ 10. The right column presents the secondary structure with lowest minimum free energy (MFE). Structure, MFE and shape probabilities determined with RNAShapes [56]. Sequence code: blue, A; green, C; red, G; yellow, U. Validated intergenic sRNAs by Northern hybridizations are **(a)** *cgb_03605* (6C RNA), **(b)** *cgb_00105* and **(c)** *cgb_20715*.

For the total set of detected *trans-*encoded sRNAs we retrieved a mean length of 90 nt (Additional file 6). To validate the sequencing approach, three sRNAs were selected and subsequently analyzed by Northern blotting: the highly conserved 6C RNA (*cgb_03605*) (Figure 5a) and two sRNAs with high read counts at their TSS (> 1000). We chose *cgb_00105* (upstream of *cg0010*) as an example of a sRNA with strong secondary structure conservation as shown above (Table 2; Figure 5b) and *cgb_20715* (downstream of *cg2071*) as an example lacking secondary structure conservation (Figure 5c). Secondary structures of all presented sRNAs were determined by minimum free energy folding and RNA shape analysis [56] which achieved high shape probabilities (~90%) in all cases, respectively. Here, the total RNAs obtained from different growth conditions were analyzed separately to monitor stress-specific transcription patterns. The sizes of these sRNAs determined by Northern blot are as follows: ~100 nt for 6C RNA, ~130 nt for *cgb_00105,* ~90 nt and ~70 nt for *cgb_20715*. In each case, the Northern blot signal of the longest sRNA correlated well with the length determined by sequencing. Compared with the Rfam prediction, the 6C RNA had a 27 nt 5′-extension (Figure 5a). This extended 5′-region seems to be conserved in the *Corynebacterium* species, since a longer 5′-region for 6C RNA was also predicted by our RNAz approach (data not shown).

The 6C RNA showed no change in transcript abundance in the Northern blots (Figure 5a). Probably due to a regulatory mechanism, *cgb_00105* appeared to be absent under heat stress conditions (Figure 5b). The sRNA *cgb_20715* also does not appear to be differentially transcribed at a tested condition. Interestingly, the Northern blot revealed a shorter second band of at ~70 nt, indicating a second RNA species possibly generated by RNA processing, since no additional promoter was observed in this region.

## Discussion

RNA sequencing is a novel approach to characterize transcriptomes of bacteria comprehensively. This technique is especially useful for detection of novel sRNAs. Here, we present the first small RNA-Seq approach for *C. glutamicum*, a member of the genus *Corynebacterium*, which represents also a model organism for the closely related genera within the *Corynebacterineae*, e.g. *Mycobacterium*, *Nocardia* etc. In comparison to the knowledge of regulatory sRNAs in the class of Gamma-Proteobacteria, especially in *E. coli* and *Salmonella* species, information about sRNAs is marginal in Actinobacteria. Until now, deep sequencing of transcriptomes in this class has only been reported for *Mycobacteria*[57, 58] and *Streptomyces* species [7].

Since sRNAs might be differentially transcribed under stress, a mixed sample of various conditions should ensure the transcription of as many sRNAs as possible. The isolation of small RNAs, however, yielded not only “true” sRNAs but also a lot of RNAs that are processed or in the process of degradation. Therefore, a number of filtering steps were performed on the cDNA reads achieved. Besides using a chosen cut-off for the increase in the number of read starts relative to the preceding position for calling a transcript start, the 5′-ends were validated by promoter searches, and from these validated 5′-ends, 3′-ends of transcripts were determined by another chosen cut-off of ten reads. It has to be stated that these cut-offs were arbitrary and adjusted to the size of the data set.

As the next step, transcripts were classified by their relative positions to annotated protein-coding sequences (CDS). The length of 5′-UTRs of coding sequences is variable, zero for leaderless transcripts and especially long for genes regulated by *cis*-regulative elements such as riboswitches. The difficulty of UTR length definition was also reported in other studies [59, 60]. Interestingly, we observed a short transcript length particularly for asRNAs. As expected, the class of leader mRNAs represents the longest transcripts, resulting from transcription into the adjacent CDS. This difference was not obtained for the different types of sRNAs in *Sinorhizobium meliloti*[60]. Generally, a shorter average size of sRNAs compared to sRNAs from other bacteria was also reported from *Streptomyces coelicolor*[7].

*Cis*-antisense sRNAs are abundant in *C. glutamicum* and located in ~15% of all annotated protein-coding genes. High-resolution tiling arrays and RNA sequencing led to the discovery of extensive antisense transcription in several other bacteria (reviewed in [18, 61, 62]). In these previous studies, the reported percentage of genes within a genome which are targeted by asRNA varies up and is >46% in *Helicobacter*[26]. The first asRNA in *C. glutamicum* was detected upstream of *cg1935*[8], thereby overlapping the mRNA of the transcriptional regulator of the GntR family in antisense direction. In our study, we detected four more asRNAs which are located opposite to already known transcriptional regulatory genes and further five asRNAs at putative regulatory genes. So far, it has been investigated that asRNAs can modulate the level of transcriptional regulators, metabolic, toxic and virulence proteins or repress transposases (reviewed in [61]). For *C. glutamicum*, our analysis of antisense transcripts based on the eggNOG classification system revealed that asRNAs seem to occur frequently at genes encoding proteins with functions in cell envelope biogenesis and protein secretion processes. However, there are hundreds of short transcripts in antisense direction of coding sequences in *C. glutamicum* the functions of which remain to be elucidated. In general, antisense transcripts often influence RNA stability of their target mRNA either by promoting or blocking ribonucleases [63–66]. Furthermore, asRNAs can induce a structural change in their target mRNA that effects transcription attenuation [67]. Other studies showed that asRNA can also hinder RNA polymerase extending the transcript encoded in the opposite strand by transcription interference (reviewed in [68]) or can affect translation of the target gene by regulation of ribosome binding [69].

Further classification, especially of *trans*-encoded sRNAs, can be done by sequence and structural analysis. The comparison of candidates predicted by the RNAz tool [20] and by the Rfam database [9] with sequencing results, allowed us to detect sRNAs that are conserved in all bacteria (housekeeping genes), in *Actinomycetales* (6C RNA) or within the closely related species *C. efficiens* and *C. diphtheriae*. However, more than the half of sRNAs seems to be specific for *C. glutamicum*. At this point we want to note that our study did not detect two widely conserved elements known to be involved in bacterial sRNA: 6S RNA [70] and short palindromic repeat (CRISPR) loci (reviewed by [71]). Interestingly, at least one CRISPR locus has been identified in the genomes of almost all other *Corynebacterium* species (CRISPRdB) [72, 73].

Many bioinformatic prediction tools were developed for sRNA research during the last decade. The comparison of the actual *in vivo* expression of sRNAs with bioinformatic prediction results often revealed only little correspondence [7, 74, 75]. Apart from the RNAz program [20] which was utilized in this study, the sRNAPredict algorithms [21, 38, 76] are prominent bioinformatic tools which have been used in various bacterial sRNA studies. As implemented in sRNAPredict, the analysis on Rho-independent terminators is often integrated in tools for sRNA detection. However, more than 75% of the *trans*-encoded sRNAs detected by our sequencing approach are not followed by a Rho-independent terminator and especially the number of asRNAs with Rho-independent terminators is marginal. A similar observation was obtained within in a search for sRNAs in *Vibrio splendidus*[77]. The correct termination of one sRNA (*cgb_00105*) at a site without an obvious terminator structure was proven by Northern blot analysis.

RNA-Seq analyses deliver an unmatched single nucleotide resolution. However, confirmatory methods are required, such as Northern blotting and are used in the present study, to look at stress-specific transcription. An example is presented with *cgb_00105*: under heat shock we detected no transcription of this sRNA whereas cold shock and chemically induced stresses had no influence on the amount of transcript. Heat shock condition was also observed to trigger a different transcription start site for ArnA *cis*-antisense RNA [8]. In the case of *cgb_20715*, two transcripts of different length are detected in each case. Here, rather sRNA maturation or degradation by endo- or exoribonucleases is likely. For 6C RNA no change by one of the chosen stress treatments was observed. At this point, there is no hint for the function of 6C RNA in *C. glutamicum.* Currently, the 6C RNA was reported to be involved in the GlxR regulatory network in *C. glutamicum*[78]. GlxR is known as a global regulator of carbon source metabolism and energy conversion. In *Streptomyces coelicolor,* 6C RNA showed an increased transcription during sporulation [79].

In bacteria there is an additional group of transcripts, comprising RNAs that act as both, regulatory RNAs and mRNAs. RNA with dual properties is exemplified by tmRNA, which combines the features of a tRNA and an mRNA. This housekeeping RNA recycles stalled ribosomes by adding a proteolysis-inducing tag to unfinished polypeptides [80]. Our results show that the tmRNA peptide-tag in *C. glutamicum* corresponds well to known sequences of a wide phylogenetic spectrum [81].

Short peptides encoded within 5′-UTRs of mRNA sequences are known as characteristic feature in a mechanism called transcriptional attenuation. In our study, we detected attenuator transcripts at different genes and operons involved in amino acid synthesis, each encoding a suitable leader peptide. Such RNAs are also included as *cis*-regulatory motives in the Rfam database. Transcriptional attenuation was first described for the tryptophan (*trp*) operon in *E. coli*[82] where terminator formation is associated to a leader sequence and is influenced by the availability of tRNA^Trp^ (RF00513). Accordingly, it has been observed for the *ilvBNC* operon in *C. glutamicum*[42] and different amino acid operons in other microorganisms (reviewed in [83]. With our analyses, we could predict further genes and operons involved in the biosynthesis of different amino acids to be regulated by the availability of uncharged tRNAs. These were detected upstream of *trpE* of the tryptophan operon, in front of the *leuA* gene (*cg0303*), encoding isopropylmalate synthase, the first step in leucine biosynthesis, and at *aroF* (*cg1129*), encoding one of the two DAHP Synthases [84] in *C. glutamicum*, responsible for the first step of shikimate pathway in the biosynthesis of aromatic amino acids. Since *C. glutamicum* is a well-known industrial producer of amino acids, these findings might become relevant for future engineering of amino acid producer strains.

## Conclusions

Our present study represents the first comprehensive screening for small RNAs in Corynebacteriaceae, a family that comprises important bacteria of industrial and medical relevance. High-throughput sequencing techniques are often applied for the search and investigation of sRNAs in bacterial genomes. Similar to sRNA studies in other bacteria, we detected hundreds of sRNA genes in *C. glutamicum* ATCC 13032. In *C. glutamicum*, more than half of all small RNAs genes was classified as antisense transcripts. C*is-antisense* sRNA genes were detected at CDS with various functions. However, CDS specifying proteins from the functional classes 'cell envelope biogenesis’ and 'secretion processes’ appear to be overrepresented. *Trans*-encoded sRNA genes were found distributed over the entire genome and showed secondary structure conservation among corynebacteria in about 30%. The 6C RNA, already known from other *Actinomycetales* genera showed strong transcription at unstressed exponential growth and all tested stress conditions. The 6S RNA, highly conserved in bacteria, was not found in *C. glutamicum*. Additionally, we detected riboswitches, transcriptional attenuators and other *cis*-regulatory motives, demonstrating the potential of our study for unraveling novel regulatory processes by small RNAs in *C. glutamicum.*

## Methods

### Preparation of cDNA libraries for RNA-Sequencing

#### Bacterial growth conditions and total RNA-isolation

*C. glutamicum* ATCC 13032 was grown in CGXII minimal medium at 30°C until exponential phase (OD_600_ 10). Cells were treated in five different stress experiments by heat (50°C), cold (4°C), diamide (N,N,N′,N′-tetramethylazodicarboxamide, 2 mM), NaCl (1.5 M), and H_2_O_2_ (0.33 M ) for 15 minutes. After harvesting 2 mL bacterial culture, pellets were resuspended in 1 mL TRIzol® reagent (Life Technologies Corporation, Darmstadt, Germany) followed by ethanol precipitation. Afterwards, crude RNA samples were treated with DNase I (Roche Diagnostics, Penzberg, Germany). After purification using phenol/chloroform/isoamyl alcohol (ratio 25:24:1), RNA was precipitated with 0.3 M sodium acetate. Purified total RNA pellets were dissolved in 50 μL RNase-free ddH_2_O. Afterwards, the purified total RNA was qualified by Agilent RNA Nano 6000 Kit on Agilent 2100 Bioanalyzer (Agilent Technologies, Böblingen, Germany).

### Preparation of two different cDNA libraries for sequencing

The purified total-RNA samples were pooled in equal parts (each condition 16 μg) and precipitated for sRNAs < 250 nt with (2.5 M sodium acetate, 25%; PEG 8000). Afterwards, rRNAs were depleted by Ribo-Zero for Gram-Positive Bacteria (Epicentre, Madison, USA). The sRNA-pool was then divided into two samples (each 5 μg). One sample (library 1) was enriched for primary transcripts by enzymatic treatment with Terminator 5′-Phosphate-Dependent Exonuclease and RNA-5′-Polyphosphatase (both enzymes from Epicentre, Madison, USA) while the second sample was prepared as whole small transcript library (library 2). The two sequencing libraries were then prepared according to the manufacturer’s instructions of TruSeq, Small RNA Kit (Illumina, San Diego, USA). Single-stranded cDNAs were created with SuperScriptII Reverse Transciptase (Life Technologies GmbH, Darmstadt, Germany). Following this, double-stranded cDNAs were generated by PCR using adapter specific primers. Afterwards, the purified libraries were quantified and qualified by Agilent High Sensitivity DNA Kit on Agilent 2100 Bioanalyzer (Agilent Technologies, Böblingen, Germany). The sequencing of the libraries was carried out at the Center for Biotechnology, Bielefeld University, utilizing the Cluster Station and the Genome Analyzer *II*x (Illumina, San Diego, USA). Each sample was sequenced on one separate lane and obtained 35 bases long single reads from the 5′-ends. Data analysis and base calling were accomplished using the Illumina instrument software.

### Bioinformatics analysis

#### Read mapping and data visualization

Reads were mapped to the *C. glutamicum* ATCC 13032 genome sequence [3] with SARUMAN [30] allowing for up to two errors per read. For the visualization of short read alignments, *Read Explorer* (Hilker *et al*., manuscript in preparation) was used. The *Read Explorer* software enables the import and visualization of a reference sequence and appropriate mapping data as so-called tracks. It is possible to scroll through the reference genome, to zoom in at each position and to look at the mapped reads at base pair level.

### Detection of transcription start sites

To automatically and systematically detect TSS, the mapping data of the library 1 enriched for primary transcripts was analyzed. First, for each strand and position of the genome, all mappings starting at the given position were counted. As possible TSS all positions on a strand were taken into account that satisfied the following criteria: for a position *i*, the number of read starts *x*_*i*_ on that strand at this position exceeded a background threshold *T* and the ratio *x*_*i*_/*x*_*i*-1_ at this position had to exceed a threshold *R*. After manual inspection of TSS, *T* was set to 19 and *R* to 5 as these parameters were found to result in a good signal to noise ratio.

### Promoter search using primary 5′-end data

Relevant stacks for promoter search were obtained from the primary transcript enriched sequencing library. For promoter search, the tool Improbizer [31, 32]) was trained with 158 published sequences from SigA binding sites [33] and 45 SigH binding sites [34], respectively. In both cases, -10 and -35 regions were correctly identified by the expectation maximization (EM) algorithm. For determination of the background significance score, control runs were performed as suggested by Improbizer. This score (mean) was used as threshold in the Improbizer runs. In each run, upstream sequences of sequencing stacks were simultaneously tested with the training set at the ratio of 1:10 which showed almost no influence on the motif search and scoring. If the -10 and -35 region motif score above the threshold and exhibit a spacer length between 16–20 bp, the test sequences were signed as TSS with indicated promoter. Since well-conserved -35-regions occur seldom in *C. glutamicum*, SigA promoters are also indicated if the well conserved extended -10 region is calculated greater than or equal the maximum of determined background significance scores, regardless of the score of the poorly conserved -35 region.

If more than one stack with indicated promoter occurred within 3 following nucleotides (278 instances), the genomic position with the strongest relative read count was selected as TSS.

### Rho-independent terminator search

The search for Rho-independent terminators in *C. glutamicum* ATCC 13032 was performed with the tool TransTermHP [39] at standard settings. Only hits with a confidence level > 0.75 were rated as Rho-independent terminators. Afterwards, terminator hits were compared with data from sequencing by search for matches within 60 nt around the assumed 3′-ends of sRNA regions.

### ORF and RBS prediction

ORF search was set at a minimum protein length of 48 nt, which is known from the leader peptide of *ilvB* transcriptional attenuator [42], up to 249 nt. The search was performed with the online tool ORFfinder by application of the following start codons: AUG, GUG, UUG and stop codons: UAA, UAG, UGA (Uhmin, Osaka University http://www.gen-info.osaka-u.ac.jp). In every case of a predicted ORF, except for leaderless transcripts, we looked for ribosome-binding sites using RBSfinder [41] applying a window size of 15 bp and the standard RBS settings (AGGAG).

### Prediction of secondary structure conservation

At first, a whole genome alignment of *C. glutamicum* ATCC 13032, *C. efficiens* YS-314 and *C. diphteriae* NCTC 13129 was created by MAUVE [85]. The search for conserved secondary structures was then performed with RNAz [20] in five different window sizes between 100–200 nt with a step size of 40 nt. According to Washietl and coworkers (2005) we minimized false positives by application of a RNA-class probability p ≥ 0.5 of the binary classification support vector machine (SVM), simultaneous with a mean pairwise identity (M.P.I.) > 60%. Afterwards, accurate tRNA and rRNA predictions were excluded yielding in 1730 hits, some of them overlapping each other. Overlapping predictions were combined and maximum RNA-class probabilities of the combined predictions were recorded, ending up with 601 loci. In total, 339 predictions showed a more stringent value of p ≥ 0.9.

### Other tools and software

RNA secondary structure analysis was performed with RNAShapes [56]. Rfam database hits for *C. glutamicum* ATCC 13032 genome were taken into account at bits scores > 90. WebLogos were created as frequency plots with the online-tool at http://weblogo.berkeley.edu. All data tables were processed with Microsoft Excel 2010, box plot diagrams were created with Origin 8.5Pro.

### Northern blot

Northern Blot analysis was performed with the total RNA isolated with TRIzol® reagent (Life Technologies GmbH, Darmstadt, Germany) obtained from different growth conditions as described above. For detection of transcripts, digoxigenin (DIG)-labeled RNA probes were produced as described in [86]. The RNA probes were synthesized with primers listed in the Additional file 7.

## Electronic supplementary material

Additional file 1: **List of mRNA leader transcripts.** (XLS 64 KB)

Additional file 2: **List of Rfam predicted** ***cis*** **-regulative motifs in** ***C. glutamicum*** **.** (XLS 25 KB)

Additional file 3: **List of cis-antisense RNAs (asRNAs and as3′-UTRs/as5′-UTRs).** (XLS 125 KB)

Additional file 4: **List of** ***trans*** **-encoded sRNAs.** (XLS 71 KB)

Additional file 5: **List of potential small mRNAs.** (XLS 34 KB)

Additional file 6: **Comparison of length distribution for asRNAs,** ***trans*** **-encoded sRNAs and mRNA leader.** The box plots display the mean (little square) and the medium (cross line) values for sRNA length. Bottom and the top of the boxes represent the 25^th^ and 75^th^ percentile, respectively, and the whiskers represent outliers. (PNG 74 KB)

Additional file 7: **Primers for Northern Blotting.** (XLS 24 KB)

## Abbreviations

RNA-Seq: High-throughput cDNA sequencing
TSS: Transcription start site(s)
UTR: Untranslated region, RBS, ribosome binding site(s)
bp: base pair(s)
nt: nucleotide(s)
OD: Optical density
×g: Times gravity
PCI: Phenol-chloroform isoamyl alcohol
PEG: Polyethylene glycol
qRT-PCR: quantitative real-time polymerase-chain-reaction
rpm: rounds per minute.
