# Supplementary figures and images for: Comprehensive discovery and characterization of small RNAs in Corynebacterium glutamicum ATCC 13032
Source: BMC Genomics. 2013 Oct 19;14(1):714. doi: 10.1186/1471-2164-14-714 (PMC4046766; doi:10.1186/1471-2164-14-714)

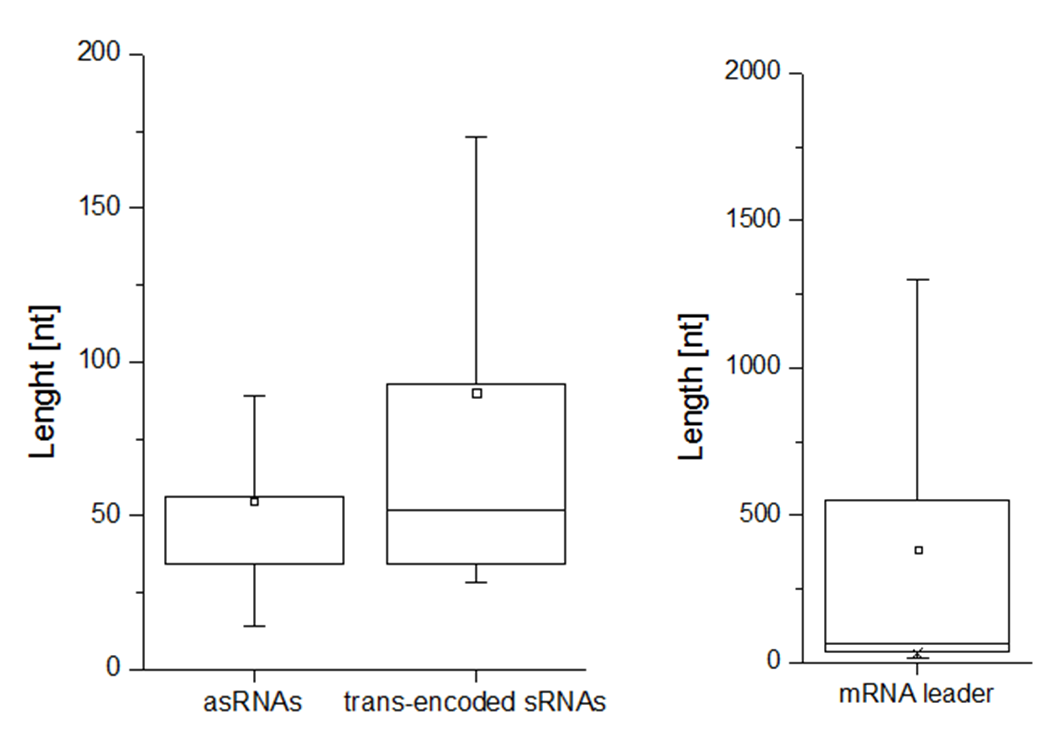

Supplement: Supplementary file 6 — Additional file 6: Comparison of length distribution for asRNAs, trans -encoded sRNAs and mRNA leader. The box plots display the mean (little square) and the medium (cross line) values for sRNA length. Bottom and the top of the boxes represent the 25th and 75th percentile, respectively, and the whiskers represent outliers. (PNG 74 KB) [file 12864_2013_5463_MOESM6_ESM.png]
